# Vibration Analysis for Fault Detection of Wind Turbines by Combining Machine-Learning Techniques and 3D Scanning Laser

**DOI:** 10.1155/2022/2093086

**Published:** 2022-12-26

**Authors:** Javier Vives, Eduardo Roses Albert, Emilio Quiles, Juan Palací, Teresa Fuster

**Affiliations:** ^1^Department of Systems Engineering and Automation, University Polytechnic of Valencia, Camino de Vera S/N, Valencia 46022, Spain; ^2^Engineering Research Team, Florida Universitària, Catarroja 46470, Spain; ^3^Red Engineering Technology Limited, Wolverton, Milton Keynes MK12 5DJ, UK

## Abstract

With this research, we apply range-resolved interferometry (RRI) to the maintenance of wind turbines using some of the most relevant machine-learning (ML) techniques. The degeneration of electrical and mechanical components of wind turbines can be predicted, detected, and anticipated using this method of automatic and autonomous learning. The vibrations in two different failure states are detected with the help of a scanner laser. In-process measurements taken by RRI agree with manual measurements, laser scanning measurements, and in-process hand measurements made following each working cycle. Consequently, the proposed method will be very useful for monitoring and diagnosing faults in wind turbines. The system will also be able to perform low-cost in-process measurements.

## 1. Introduction

The development of new techniques for maintaining wind power infrastructure in recent years has increased wind production by about 80% [1]. Monitoring and fault diagnosis can improve the reliability, safety, and profitability of a wind turbine. Traditionally, fault tree analysis and spectral analysis have been used to maintain wind turbines.

As digital and mobile technology advances, artificial intelligence is becoming more popular. In these fields, machine learning is having an even greater impact due to new hardware and cloud-based solutions [2]. The cause of vibration is usually a mechanical or electrical failure. It is also possible to detect failures of gears and bearings by vibrations. Rolling elements wear primarily because their surface position changes continuously as the load increases. Introducing new hardware and cloud-based solutions has made machine learning even more impactful in these areas [3]. Besides geometric imperfections, vibrations can also occur as a result of the failure of components, cage failures, imbalances, and misalignments. The central point of illumination and detection provided by interferometric techniques (including RRI) makes it easier to integrate them into mounting structures. As an interferometric technique similar to RRI [4–6], optical coherence tomography (OCT) is widely used in monitoring. In principle, OCT can achieve a resolution of 0.01 mm (compared to RRI's maximum working range of 10 cm). Consequently, self-referencing 3D scanners cannot be determined using OCT systems because their operating range is limited. Furthermore, typical swept-source OCT systems for monitoring processing applications cost about $200 k, which compromises the low-capital-cost advantage of surveillance and fault detection. As a result of its use of monolithic laser diodes and fibre components that are less expensive than traditional systems, RRI's OCT system compares favourably against conventional systems, which utilize expensive laser sources. Machine learning works perfectly with AI [7]. There are some limitations to this type of methodology, as well as some drawbacks. Maintenance methodologies allow automatic detection, detection, and categorization of malfunctioning functions. Machine learning reduces response times, reduces errors, and allows for flexible offshore implementation and feedback learning through the use of data management and analysis. To implement AI on a real system without causing costly errors, the method must be validated. With AI methodologies, all types of failures can be analysed and prevented [8]. The use of prototypes and test benches is helpful whenever you are developing new techniques, conducting studies, and so on. When validating fault diagnosis techniques, it is also helpful to understand how these systems work. Broken wind turbines are expensive to replace, as well as losing energy, since they cannot produce power during peak times.

In offshore wind farms with expensive repair and maintenance costs, detecting and diagnosing faults early is essential if the machine is to be stopped [9]. To reduce downtime and defective product costs, maintenance activities must be managed efficiently. Our prototype detects failures, supervises them, and anticipates them based on algorithms that anticipate and prevent problems. The study presents a method for monitoring and diagnosing faults in a prototype wind turbine using vibration analysis and RRI techniques. Different bearing failure types can be detected automatically using the algorithm presented here. Data collection and analysis were followed by a comparison of the results after a literature review. The study ends with several conclusions.

## 2. Research Methodology

In turbine bearings, vibration can be detected and monitored with different methods based on the bearing's characteristics. Because of this, the characteristics of the bearing may not always match the characteristics of the fault. Through machine learning and laser scanning, we demonstrate how vibration measurements from another bearing can improve accuracy and predict failure.

### 2.1. Artificial Intelligence

In wind turbine fault detection, machine-learning techniques focus on detecting anomalous behaviour and classifying faults. Additionally, this technique enables a quick response to failures or anticipated problems, enhancing both the performance and the security of the system. Most machine-learning methods are supervised [10]. The output of supervised learning is already known. Unsupervised learning has no known outcome. Incoming and outgoing processes are the same. In contrast to supervised learning, only binary logic is required for unsupervised learning. There is no use of references. Data must first be classified before any type of learning can be applied. The solution to this problem can be achieved by using a variety of classification algorithms. For a wind turbine, *k*-nearest neighbor (KNN) and support vector machine (SVM) are two of the most important algorithmic classifiers [11].

### 2.2. Support Vector Machines

**Figure 1:**
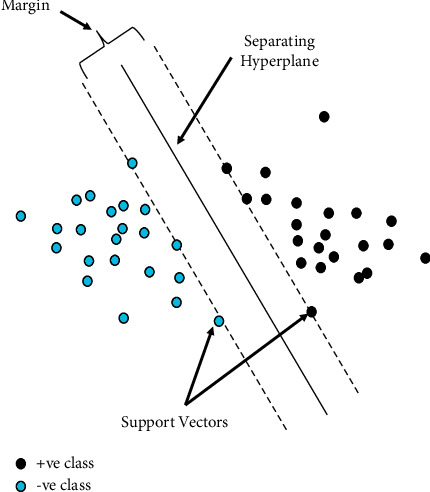
Optimal hyperplane for binary classification by an SVM.

An SVM is a machine-learning algorithm based on statistical learning theory. This method works well for classification and regression, such as in fault diagnosis, when we use small samples. It is shown that a linear classifier can separate two simple classes. These two types of samples are represented by triangles and squares in Figure 1. Two classes can be separated by a hyperplane H. In these two classes, the planes H_1_ and H_2_ (shown in dashed lines) are parallel to H and pass-through samples that are closest to H. Margins are calculated by taking the distance between H_1_ and H_2_. In the SVM, linear boundaries are placed between two distinct classes H_1_ and H_2_. The margin is maximized, so the generalization error is the smallest. Support vectors are often used to measure margins, and they include the closest points to the margin [12]. A quadratic function is minimized under linear inequality constraints by reducing it to convex optimization [12]. Assume that we have a training set of samples [(*x*_*i*_,(*y*_*i*_)], where *i* = 1 to *N*, and *N* represents the total number of samples. To find the separation plane with the least generalization error out of each linear separation plane, we need to determine how to divide the input samples into two classes. It is possible to divide the samples into two classes, i.e., triangular and square. A triangle class has a (*y*_*i*_=−1 label. A square class has a *y*_*i*_=+1 label. For nonseparable data, slack variables are not considered (nor P 0). Using the following optimization problem, you can obtain the hyperplane for *f* (*x*) = 0 from the given data:

(1) $$\begin{array}{c} Minimize{\frac{1}{2}\left|\left|w\right|\right|}^{2}+C\overset{N}{\underset{i=i}{\sum }}\xi_{i}\text{,}\\ Subject\;to\left\{\begin{array}{c} y_{i}\left(w^{T}x_{i}+b\right)\geq 1-\xi_{i}\text{,}\\ \xi_{i}\geq 0,i=1,2,\ldots N\text{,} \end{array}\right. \end{array}$$

where *C* is a constant representing the error penalty. Introducing Lagrange multipliers to the optimization problem above leads to the following result:

(2) $$\begin{array}{c} Minimize\;W\left(\lambda \right)=\overset{N}{\underset{i=i}{\sum }}\lambda_{i}-\frac{1}{2}\overset{N}{\underset{ij=i}{\sum }}y_{i}y_{j}\lambda_{i}\lambda_{j}\left(x_{i}x_{j}\right)\text{,}\\ Subject\;to\left\{\begin{array}{c} 0\leq \lambda_{I}\leq C\text{,}\\ \overset{N}{\underset{i=i}{\sum }}\lambda_{i}y_{i}=0,i=1,2,\ldots ,N, \end{array}\right. \end{array}$$

There are several advantages and limitations to AI classifiers, as shown in Table 1.

The use of machine learning has recently demonstrated state-of-the-art performance in areas such as computer vision, audio recognition, and fault diagnosis [13], and support vector machines (SVM) and *k*-nearest neighbour (KNN) have been used to diagnose rotating machinery faults. AI classifiers are compared in Table 2.

### 2.3. Range-Resolved Interferometry (RRI)

Monolithic laser diodes are a more cost-efficient alternative to OCT that can be utilized with RRI via different processing algorithms. It is described in [14, 15] how RRI works. As part of this technique, a diode laser is modulated with a sinusoidal optical frequency, and reflected light is delivered from a target (layer surface), and the light reflected from a fibre tip is interfered. Using a smooth window function to demodulate an interference signal gives a sinusoidal signal with a frequency indicating how far away the target was from the fibre tip and amplitude indicating the intensity of the reflection. Whenever sinusoids whose amplitudes exceed a certain threshold are recorded, their centre position relative to the fibre tip position is measured.

### 2.4. Implementation of Scanned RRI

**Figure 2:**
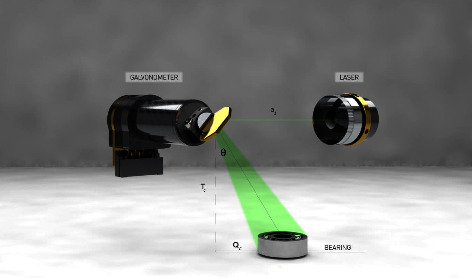
Scanned range-resolved interferometry for 3D implementation.

Following the methodology, study, and design carried out by [6], RRI's instrument provides a 4.8 kHz data rate. In Figure 2, the data output is the galvanometer scan angle and the signal amplitude (corresponding to the distance along the laser beam from the fibre tip at each instance). According to [6] the RRI head unit can convert polar coordinates into Cartesian coordinates using the geometrical relations given in equations (3)–(5) using the distance between the galvanometer mirror and the reflection and the galvanometer angle:

(3) $$\begin{array}{c} Q_{C}=\left(a-a_{l}\right)\cos\;\theta \text{,} \end{array}$$

(4) $$\begin{array}{c} W_{C}=\left(a-a_{l}\right)\sin\;\theta \text{,} \end{array}$$

(5) $$\begin{array}{c} t_{C}=0\text{.} \end{array}$$

In addition to the galvanometer angle, the distance between the galvanometer and the reflection *d*, as well as the distance between the galvanometer and the fibre tip *a*_*l*_ determines the galvanometer angle *θ*. It is possible to accurately calibrate the galvanometer length by turning the mirror directly back towards the fibre tip with the RRI instrument. According to [6] if the galvanometer mirror angle *θ* is zero and the beam is angled vertically, then the equation describes a two-dimensional case in which components on the *y*-axis are absent (i.e., the 3D diagram shown in Figure 2). Thus, the RRI instrument outputs a point cloud that describes reflections occurring at specific spatial locations and times *q*_*c*_, *w*_*c*_, *t*_*c*_, each given by an array of values [6].

## 3. Case Study

Detailed information is provided regarding the industrial environment, the components, and the location of the laser within the system in the report. We also explore a method of acquiring data.

### 3.1. Prototype

**Figure 3:**
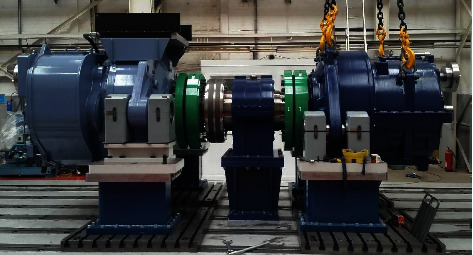
Wind turbine test bench.

Figure 3 illustrates how a small wind turbine's parts wear and deteriorate, along with its effects [16]. By testing diagnostic techniques before deterioration occurs, parts can be replaced before deterioration occurs. It is possible to install the scanning laser at different locations on the prototype wind turbine. It is possible to position the scanning laser in each stage of the multiplier based on state monitoring techniques and machine design. Input bearings can be monitored with a laser for vibrations caused by fast shaft coupling to generators. There will be a propagation of the signal between the stages, and various failures will affect the vibrations. On the slow axis, there is also an interesting bearing for measuring the prototype. It is possible to replace this element in some damaged bearings to determine how the signal behaves after failure as well as how the bearing itself deteriorates over time. For this research, we have used the Thorlabs GVS005 Galvo scanner and an adjustable collimator.

### 3.2. Data Collection

The galvo scan occurred 90 seconds after calibration, corresponding to a position along the *y*-axis of 1848 mm. We measured 7.8 *θ* angular amplitude with an angular frequency of 28 Hz and 3.9 kHz data rate. The RRI instrument falls below detected noise levels on steeper sides due to too much light reflection [6]. Because the angled sidewalls scatter more light, the RRI instrument will have better coverage of the sidewalls for materials with less specular reflection. Python scripts are used to control the measurements of RRI instruments, and they need to be started manually each time a measurement is conducted.

## 4. Results

**Figure 4:**
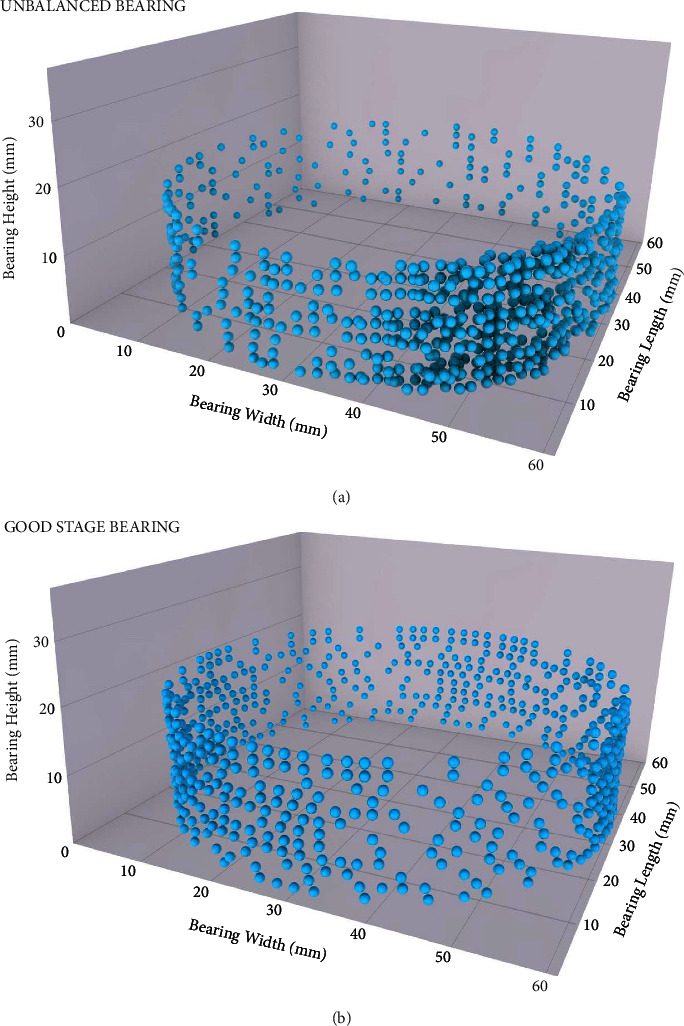
(a) Imbalance: predicted output algorithm. (b) Good stage: predicted output algorithm.

The simulation is successfully run using the laser scanning data provided in the previous section. From 0 to 1500 rpm, the prototype is capable of rotating at five different speeds. The medium speed in this case was 300 rpm. Wind turbine failures can be tracked, diagnosed, and prevented with automated learning systems. Based on an average of 5000 samples generated by a scanning laser, a 3.9 kHz graphical presentation was generated. In addition to tracking, preventing, and diagnosing wind turbine failures, automated learning systems can also predict them. When an algorithm is properly trained, it can analyse and categorise data independently after receiving feedback, enabling it to correctly predict the future. A good stage and an imbalance were simulated during the simulation. The data were acquired using a galvo-laser scanner, then filtered and then processed, resulting in four phases of analysis. It is not possible to analyse a signal generated at random. The conditioning and processing of machine-learning algorithms are essential for extracting patterns from signals of this type. Furthermore, the signal is hard to analyse and learn from because of its time variations. First, the algorithm must be filtered and conditioned to ensure that it will work correctly. A signal processing algorithm reads the invariant characteristics of a signal in time. Identifying faults and conditions requires the extraction of features. Arithmetic means are calculated based on the number of tests and examples of each problem. A principal component analysis is then used to reduce the dataset to the minimum number of variables necessary to represent the original variables. Moreover, we can determine the standard deviation for each of the stipulated failure conditions to make future decisions based on a better understanding of the current state. Compared to the average, there are many dispersed states in the data, which means the model should work. Each one-off simulation will be defined and explained. First, the mathematical processes explained above are used to generate the two simulated states. An imbalance can be seen as in Figure 4(a). This machine-learning algorithm consists of a few interesting aspects to be defined. An imbalance in the wind turbine prototype is perfectly illustrated by the plot obtained from Python scripts. Some points appear to have been misinterpreted as noise by the 3D laser scanner postprocessor. Considering the limits we have used in this case, the imbalance algorithm may be outdated. The bearing stage shown in Figure 4(b) is a good one. Scanner laser data are very stable over time. There are over 5000 different points presented in total. The algorithm sometimes makes a mistake in predicting the good stage when there is nothing, but unknowable factors are involved.

In both cases, the data can be grouped well by combining AI algorithms with laser scanning software. Stages are classified and analysed correctly. No matter what the failure conditions are (imbalance or good stage), the algorithm produces highly accurate predictions.

The combination of artificial intelligence and 3D scanner lasers is therefore considered to have great potential for future development in the maintenance sector, as our wind turbine prototype worked well with the variables, simulations, and results we considered, allowing us to accurately forecast failures.

## 5. Conclusions

For AI to be successful and operate properly, data must be acquired and classified. The combination of scanning lasers and machine-learning systems makes it easier to detect, monitor, and diagnose faults in wind turbines. Wind turbine bearing failures are diagnosed and prevented through vibration analysis by combining both technologies. With the RRI instrument, it is easier to supervise in-process activities and diagnose failures. There were no significant differences in quality between results obtained before and after processing. Up to a scanning angle of 4 degrees, galvanometer measurements of RRI provide good coverage of the bearing.

By combining AI and scanning lasers, bearing faults can be diagnosed, which has the advantages such as robustness, high accuracy, and high processing speed, making it very suitable for this type of study. Due to the method's effectiveness, it can be applied to other mechanical components of wind turbine prototypes to identify or prevent potential failures. A prototype can be used to study, develop, and validate fault diagnosis and supervision techniques, as well as replace worn or defective parts with new ones. Tests are conducted on prototype wind turbines to evaluate diagnostic algorithms that are going to be installed in high-performance turbines. As a result, cost and time savings are achieved, and algorithms can be verified, adjusted, and corrected.

## Figures and Tables

**Table 1:** Artificial intelligence: advantages and limitations.

Algorithm	Advantages	Limitations
SVM	High sorting accuracy Can deal with high-dimensional features	Low efficiency for big data No physical meaning
Deep learning	Automatic fault recognition and learning features It does not need the function extractor	Large sample needs No physical meaning A lot of training

**Table 2:** Performance comparison.

	SVM	Deep learning
Sorting speed	*∗∗∗*	*∗*
Overall accuracy	*∗∗∗*	*∗∗∗*
Noise robustness	*∗*	*∗∗∗*
Overfitting	*∗*	*∗∗*
Robustness to parameters	*∗*	*∗∗*
Physical explanation	*∗*	*∗*

## Data Availability

The data generated or analysed during this study are included within the article.
